# Bi-specific molecule against EGFR and death receptors simultaneously targets proliferation and death pathways in tumors

**DOI:** 10.1038/s41598-017-02483-9

**Published:** 2017-06-01

**Authors:** Yanni Zhu, Nicole Bassoff, Clemens Reinshagen, Deepak Bhere, Michal O. Nowicki, Sean E. Lawler, Jérémie Roux, Khalid Shah

**Affiliations:** 1 Center for Stem Cell Therapeutics and Imaging, Massachusetts General Hospital, Harvard Medical School, Boston, Massachusetts 02114 USA; 2 Center for Stem Cell Therapeutics and Imaging, Brigham and Women’s Hospital, Harvard Medical School, Boston, Massachusetts 02115 USA; 3 Department of Radiology, Massachusetts General Hospital, Harvard Medical School, Boston, Massachusetts 02114 USA; 4 Department of Neurology, Massachusetts General Hospital, Harvard Medical School, Boston, Massachusetts 02114 USA; 5 Department of Neurosurgery, Brigham and Women′s Hospital, Harvard Medical School, Boston, Massachusetts 02115 USA; 6 Department of Systems Biology, Harvard Medical School, Boston, Massachusetts 02115 USA; 7 Harvard Stem Cell Institute, Harvard University, Cambridge, Massachusetts 02138 USA

## Abstract

Developing therapeutics that target multiple receptor signaling pathways in tumors is critical as therapies targeting single specific biomarker/pathway have shown limited efficacy in patients with cancer. In this study, we extensively characterized a bi-functional molecule comprising of epidermal growth factor receptor (EGFR) targeted nanobody (ENb) and death receptor (DR) targeted ligand TRAIL (ENb-TRAIL). We show that ENb-TRAIL has therapeutic efficacy in tumor cells from different cancer types which do not respond to either EGFR antagonist or DR agonist monotherapies. Utilizing pharmacological inhibition, genetic loss of function and FRET studies, we show that ENb-TRAIL blocks EGFR signalling via the binding of ENb to EGFR which in turn induces DR5 clustering at the plasma membrane and thereby primes tumor cells to caspase-mediated apoptosis. *In vivo*, using a clinically relevant orthotopic resection model of primary glioblastoma and engineered stem cells (SC) expressing ENb-TRAIL, we show that the treatment with synthetic extracellular matrix (sECM) encapsulated SC-ENb-TRAIL alleviates tumor burden and significantly increases survival. This study is the first to report novel mechanistic insights into simultaneous targeting of receptor-mediated proliferation and cell death signaling pathways in different tumor types and presents a promising approach for translation into the clinical setting.

## Introduction

Epidermal growth factor receptor (EGFR) is an oncogenic driver in many human cancers^1–3^ and EGFR amplification as well as EGFR activating mutations are associated with poor prognosis and decreased patient survival. Consequently, EGFR is considered as one of the most important molecular targets in clinical oncology. Currently available EGFR targeting agents include small-molecule receptor tyrosine kinase inhibitors (RTKI)^4^ and monoclonal antibodies (mAb) such as Cetuximab^5^. However, the intrinsic and acquired resistance to EGFR inhibitors in tumor cells has become a significant challenge in EGFR-targeted therapies. For example, KRAS mutations and activation of HER2 are significantly associated with resistance to Cetuximab therapy in colorectal cancer patients^6, 7^. Furthermore, the EGFR secondary mutation T790M^8^ and MET amplification^9^ have been shown to play a major role in the resistance of lung cancer cells to small molecule EGFR inhibitors. Activation of cell death pathways in tumor cells using death receptor agonists has been identified as a promising approach in many cancer types and is currently in clinical trials^10^. However, the varying response of tumor cells to DR agonist-mediated apoptosis in different cancer types has limited their use in a broader spectrum of tumors^11–13^. Thus there is an urgent need to develop and characterize tumor specific agents to overcome these resistance mechanisms. Simultaneous targeting of both cell death and proliferation pathways offers a strong rationale to achieve therapeutic efficacy in a broad spectrum of cancer patients unresponsive to mono-therapeutic approaches.

Bi-specific antibodies targeting EGFR and other receptor tyrosine kinases (RTKs) have been shown to improve outcomes in comparison to monospecific therapy in certain cancer contexts^14, 15^. Moreover, molecular targeting of EGFR with antibody conjugates such as anti-EGFR human Fab-Taxol^16^, or boronated EGFR mAb^17^ have shown some efficacy against EGFR driven tumors. However, none of these EGFR conjugate studies has addressed whether these agents are effective in cancers, which are inherently non-responsive to individual or combinational therapies and whether anti-tumor efficacy is altered in tumor cells which harbor oncogenic mutations downstream of EGFR.

Nanobodies (Nbs) are derived from heavy chain-only antibodies found in camelids (e.g. llama), and consist solely of the antigen binding domain (VHH)^18^. Recently, Nbs specific to cancer-related antigen such as EGFR^19^, hepatocyte growth factor (HGF)^20^ or chemokine receptor CXCR7^21^ have been isolated, and Nb-based anti-cancer therapies have been emerging^22^. Llama glama-derived EGFR-specific nanobodies (ENbs) have been shown to sterically hinder receptor-ligand binding, thereby inhibiting EGFR downstream signalling. In an effort to allow simultaneous targeting of multiple surface receptor-mediated pathways, we have previously engineered bivalent ENbs and shown their efficacy in mouse tumor models^23^. In this study, we extensively studied the molecular mechanism of ENb-TRAIL mediated cell killing in a broad spectrum of colorectal cancer, lung cancer, and glioma cell lines non-responsive to EGFR and death receptor targeted therapies. In order to translate our findings into clinical settings, we further explored the potential of using synthetic extracellular matrix (sECM) encapsulated stem cells expressing ENb-TRAIL in a mouse tumor model of glioblastoma resection which mimics the clinical scenario of glioblastoma tumor growth and resection.

## Results

### ENb-TRAIL induces apoptosis in cancer cells unresponsive to EGFR and DR targeted mono-therapies

**Figure 1:**
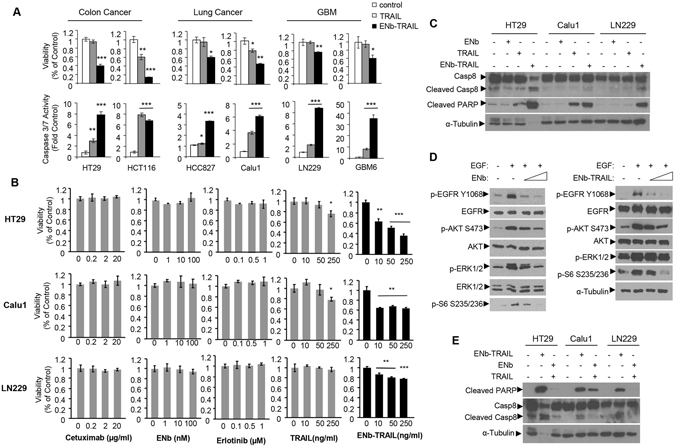
ENb-TRAIL has superior efficacy in cancer cells resistant to EGFR-targeted therapy and DR agonist TRAIL *in vitro*. (**A**) Cell viability and caspase 3/7 activity of tumor cells from different cancer types in response to 24 h treatment with TRAIL (50 ng/ml) or ENb-TRAIL (50 ng/ml). (**B**) Cell viability of HT29, Calu1 and LN229 cells in response to 24 h treatment with Cetuximab, ENb, Erlotinib, TRAIL or ENb-TRAIL. (**C**) Western blot showing the induction of apoptosis in tumor cells 24 h-post treatment with ENb-TRAIL (50 ng/ml) as compared to ENb (100 nM) or TRAIL (50 ng/ml). (**D**) Western blot analysis of EGFR signaling in ENb or ENb-TRAIL treated LN229 cells. (**E**) Western blot analysis showing the induction of apoptosis in ENb-TRAIL vesus the combination of ENb and TRAIL treated tumor cells (post 24 h treatment). *P < 0.05, **P < 0.005 and ***P = 0.0001 determined by unpaired *t* test. Error bars indicate SD. Western blots were cropped to show specific bands only. For uncropped blots see Fig. S11.

ENb-TRAIL consists of cDNA fusions encoding bivalent and bispecific EGFR targeted nanobodies (ENbs) and a potent cytotoxic variant of TRAIL (Fig. S1A). To investigate the wide range of therapeutic efficacy of ENb-TRAIL in different types of tumors, we tested ENb-TRAIL on a panel of EGFR amplified, mutated or wild type colon (HT29, HCT116), lung (Calu1, HCC827), and glioma (LN229, GBM6) cell lines with varying levels of EGFR, DR4 and DR5 expression levels (Fig. S1B,C). A significantly reduced cell viability associated with increased caspase 3/7 activity was observed in all six cell lines treated with a low dose of ENb-TRAIL as compared to TRAIL and control (Fig. 1A). To understand the mechanism of ENb-TRAIL function, we compared the efficacy of ENb-TRAIL with EGFR-targeted therapeutics (monoclonal antibody Cetuximab, ENb or Erlotinib) or with TRAIL alone at different doses in HT29 (colon), Calu1 (lung), and LN229 (glioma) tumor lines. Monotherapy with both EGFR binding antibodies (Cetuximab, ENb) as well as with the EGFR-specific tyrosine kinase inhibitor, Erlotinib did not influence the cell viability of the cell lines tested (Fig. 1B). However treatment with Erlotinib resulted in inhibition of EGFR and pAKT activity (Fig. S2). Similarly all the tumor cell types were unresponsive to TRAIL-mediated apoptosis (Fig. 1B). In contrast, ENb-TRAIL treatment significantly reduced cell viability in a dose dependent manner (Fig. 1B) and resulted in apoptosis mediated by caspase 8 cleavage, and subsequent cleavage of PARP (Fig. 1C). These results demonstrate that ENb-TRAIL induces caspase-mediated apoptosis in tumor cells that are unresponsive to EGFR and DR-targeted therapy.

To test whether the apoptotic effect of ENb-TRAIL is simply through simultaneous targeting of EGFR and DR pathways, we compared the efficacy of ENb-TRAIL with the combination of EGFR blockade and TRAIL. Western blot analysis showed that both ENb and ENb-TRAIL significantly reduced ligand-dependent activation of EGFR and its downstream effectors PI3K/AKT, MAPK and mTOR/ribosomal S6 (Fig. 1D). However, ENb-TRAIL treatment was much more efficient in inducing DR-mediated apoptosis as compared to combined treatment with ENb plus TRAIL in TRAIL insensitive HT29, Calu1 and LN229 cells (Figs 1E and S3A,B). Moreover, pretreatment with Erlotinib prior to TRAIL or ENb-TRAIL treatment did not influence the viability of HT29 and LN229 tumor cells (Fig. S3C). Together, these results show that ENb-TRAIL blocks EGFR activity as effectively as ENb, however, ENb-TRAIL mediated induction of apoptosis is not recapitulated by the combination treatment of EGFR inhibition and TRAIL. These results indicate that ENb-TRAIL is directly involved in activating DR signaling in addition to blocking EGFR and priming tumor cells for DR mediated apoptosis.

### ENb-binding to EGFR is critical for ENb-TRAIL activation of apoptosis

**Figure 2:**
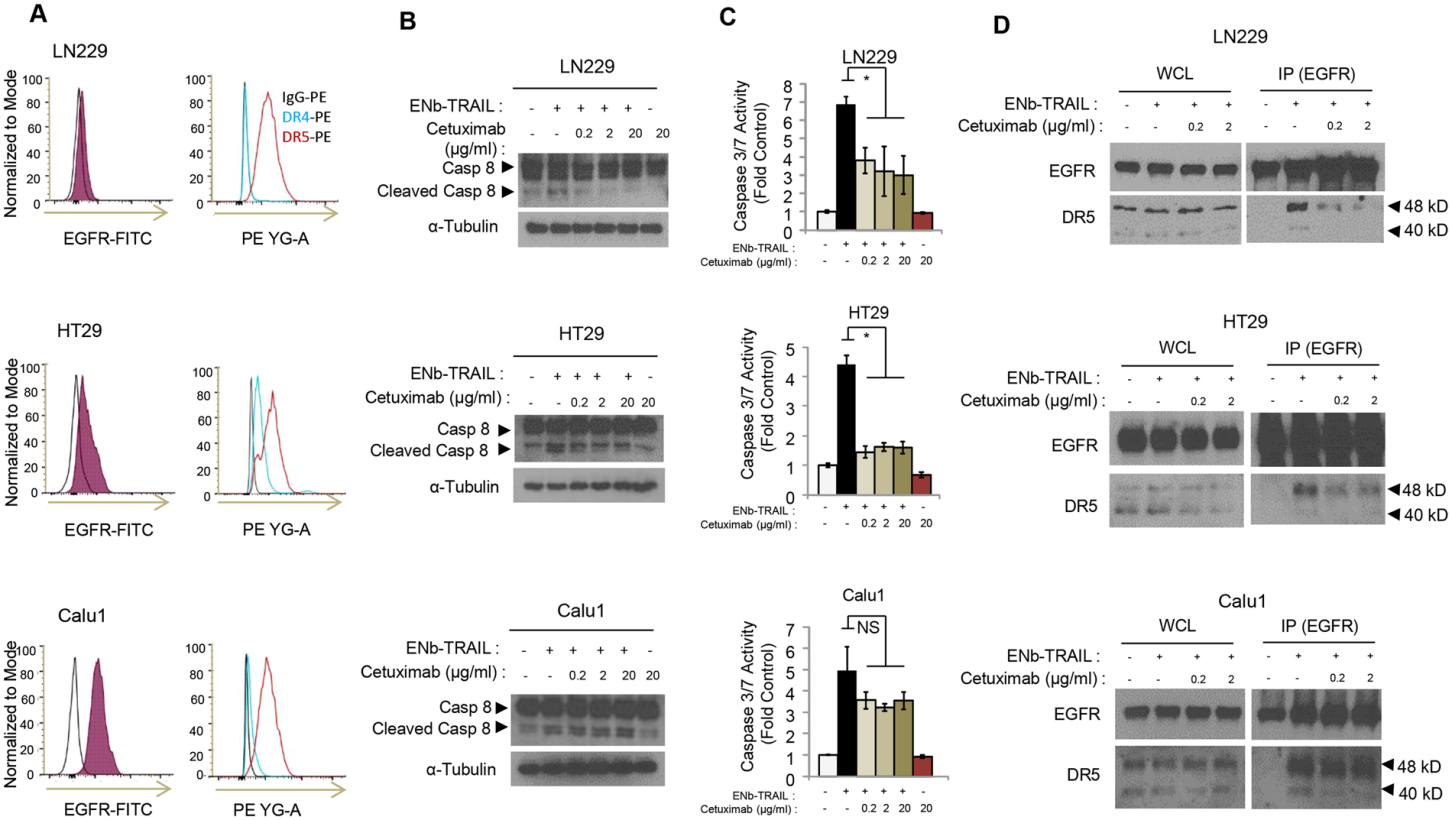
ENb-binding to EGFR is critical for ENb-TRAIL activation of apoptosis. (**A**) Differential cell membrane EGFR, DR4, and DR5 expression levels in LN229, HT29 and Calu1 cells measured by Flow Cytometry. Left panel: cell membrane EGFR expression. Right panel: cell membrane DR4 and DR5 expression. (**B–C**) Cells were pretreated with Cetuximab for 30 min and then treated with ENb-TRAIL for 8 h and apoptosis markers were analyzed by Western blotting (**B**) and caspase 3/7 assay (**C**). *P < 0.05 determined by unpaired *t* test. Error bars indicate SD. (**D**) Co-immunoprecipitation and Western blot analysis showing EGFR and DR5 complex formation in the presence of ENb-TRAIL and the attenuation of complex by Cetuximab. Western blots were cropped to show specific bands only. For uncropped blots see Fig. S12.

To assess the superior function of ENb-TRAIL over the combination of ENb and TRAIL, we next investigated the additional role of ENb in EGFR signaling. Flow cytometry analysis showed that all three lines had similar cell surface DR5 expression levels, whereas LN229 cells showed a low level cell surface EGFR and almost no cell surface DR4 expression compared to HT29 and Calu1 (Fig. 2A). These data suggested that DR5 might play a more important role than DR4 in ENb-TRAIL induced apoptosis. Next, we compared ENb with EGFR monoclonal antibody Cetuximab to block ENb-TRAIL binding to EGFR. Both ENb and Cetuximab are known to target the extracellular domain III of EGFR^19, 24, 25^, therefore Cetuximab should compete with ENb-TRAIL binding to EGFR. Western blot analysis of cleaved caspase-8 and caspase 3/7 activity assays revealed that the pre-treatment with Cetuximab or ENb were comparable and significantly reduced ENb-TRAIL induced apoptosis in all the three tumor lines tested (Figs 2B,C and S4). To further investigate the role of EGFR binding in apoptosis induction post ENb-TRAIL treatment, we performed co-immunoprecipitation assays to evaluate changes in EGFR and DR5 interaction in the presence of ENb-TRAIL and Cetuximab. EGFR and DR5 formed a complex in the presence of ENb-TRAIL in all three lines. Pre-treatment with Cetuximab significantly reduced ENb-TRAIL-induced apoptosis in LN229 and HT29 cells but this apoptosis inhibition was not to the same extent in Calu1 cells. The reduced apoptosis inhibition in Calu1 was correlated with the reduced blocking of EGFR-ENb-TRAIL-DR5 complex by Cetuximab (Fig. 2D and S5A). These results indicate that ENb-binding to EGFR is critical for *EGFR*-ENb-TRAIL-*DR5* complex formation and ENb-TRAIL induced activation of the caspase cascade in ENb and TRAIL insensitive tumor cells.

### DR5 plays a major role in ENb-TRAIL mediated apoptosis

**Figure 3:**
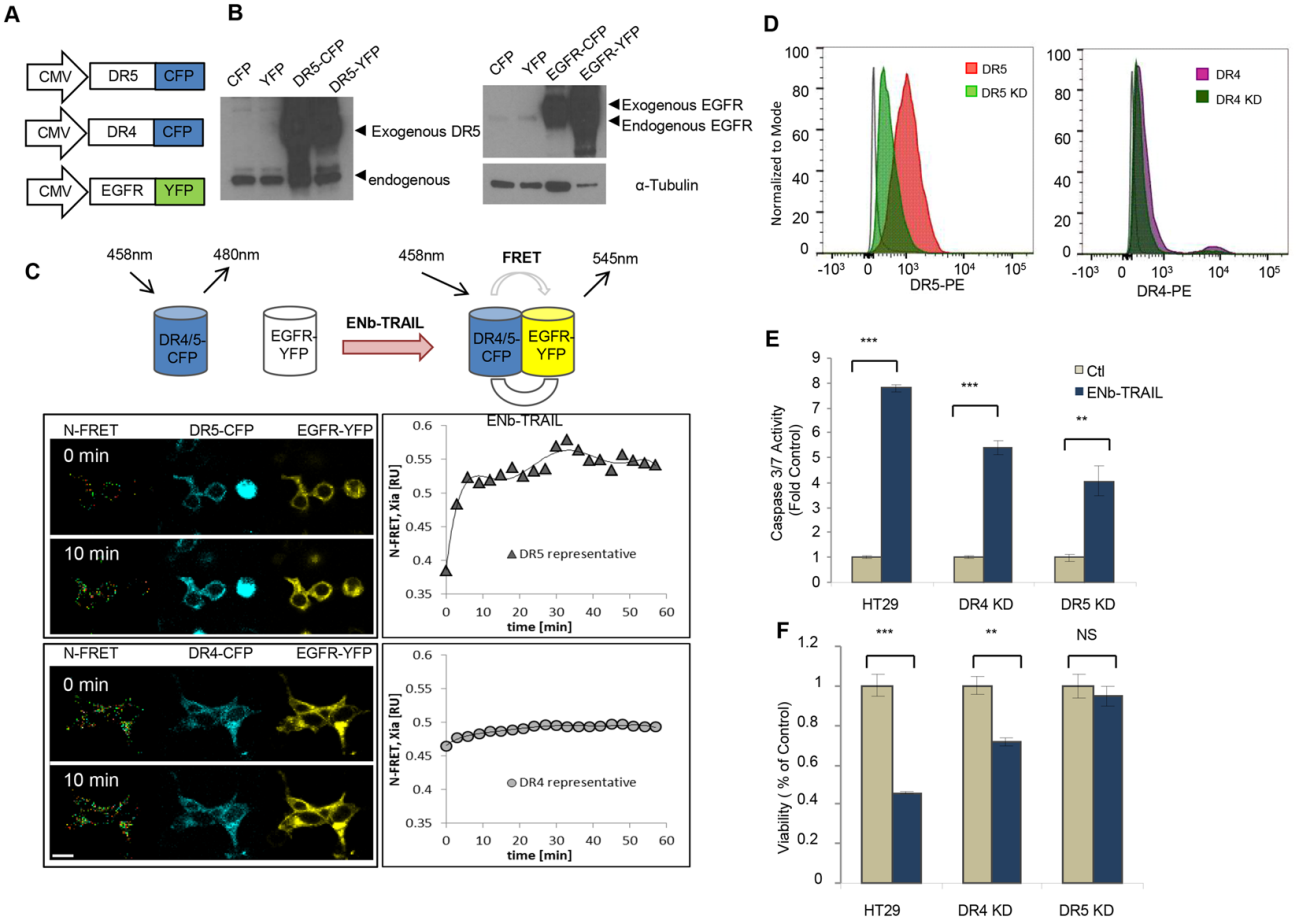
TRAIL receptor DR5 plays a major role in ENb-TRAIL-induced apoptosis. (**A**) Schematic showing DR4/5-CFP and EGFR-YFP fusion protein constructs. (**B**) Western blot analysis of DR5-CFP/YFP and EGFR-CFP/YFP expression in 293 T cells. (**C**) Changes in FRET signal between DR4/5-CFP and EGFR-YFP post treatment with ENb-TRAIL in 293 T cells. The left panels show frames from time lapse collection of DR5-CFP or DR4-CFP + EGFR-YFP signals and calculated N-FRET index (Xia); index is not calculated for overexposed signal. Right panel: The quantification of N-FRET index over 1 h period. Cells were treated with ENb-TRAIL at time 0 min. (**D**) Flow cytometry analysis of membrane DR5 or DR4 level in knocked down (DR5 KD, DR4 KD) HT29 cells. (**E**) Caspase 3/7 activity assay and (**F**) cell viability assay analysis of DR5 or DR4 KD effect on ENb-TRAIL induced apoptosis. *P < 0.05, **P < 0.005 and ***P = 0.0001 determined by unpaired *t* test. Error bars indicate SD. Western blots were cropped to show specific bands only. For uncropped blots see Fig. S12.

To understand the dynamics of EGFR and DR5 on tumor cells post ENb-TRAIL binding and subsequent activation of DR5, we engineered fusion constructs EGFR-YFP, DR4-CFP and DR5-CFP and co-expressed EGFR-YFP and DR5-CFP or DR4-CFP in 293T cells (Figs 3A,B and S5B). We then tested whether binding of ENb-TRAIL led to receptor clustering and FRET between EGFR-YFP and DR5-CFP or DR4-CFP (Fig. 3C). Time-lapse acquisition of FRET analysis showed that transient expression of EGFR-YFP and DR5-CFP displayed a basal level of FRET signal and treatment with ENb-TRAIL led to a rapid increase in the FRET signal accompanied by DR5-CFP clustering in 293 T (Fig. 3C; upper panel). In contrast, there was no increase in FRET signal between EGFR-YFP and DR4-CFP upon ENb-TRAIL treatment (Fig. 3C; lower panel). To test whether DR4 also functions in ENb-TRAIL induced apoptosis, we introduced DR4-CFP into EGFR and TRAIL insensitive LN229 cells that have minimal levels of membrane DR4 (Fig. 2A). The overexpression of DR4-CFP in LN229 cells increased the basal level of apoptotic cells measured by caspase 3/7 activity but had no significant effect on ENb-TRAIL mediated induction of apoptosis compared to overexpression of LN229-CFP cells (Fig. S6). These data imply that DR5 and EGFR are co-localized in the plasma membrane and binding of ENb-TRAIL results in EGFR and DR5 receptor clustering. Next we evaluated whether shRNA knock down of either DR4 or DR5 influences ENb-TRAIL induced apoptosis. Stable expression of DR4 or DR5 shRNA in HT29 cells resulted in a significant reduction in the membrane expression of DR4 and DR5 respectively (Fig. 3D). Cell viability and caspase 3/7 assays showed that the reduction of membrane DR5 and to a lesser extent DR4 significantly reduced ENb-TRAIL mediated apoptosis (Fig. 3E,F). The results suggest that DR5 plays a major role in ENb-TRAIL induced apoptosis.

### Therapeutic stem cell delivered ENb-TRAIL has anti-tumor effects *in vitro* and *in vivo*

**Figure 4:**
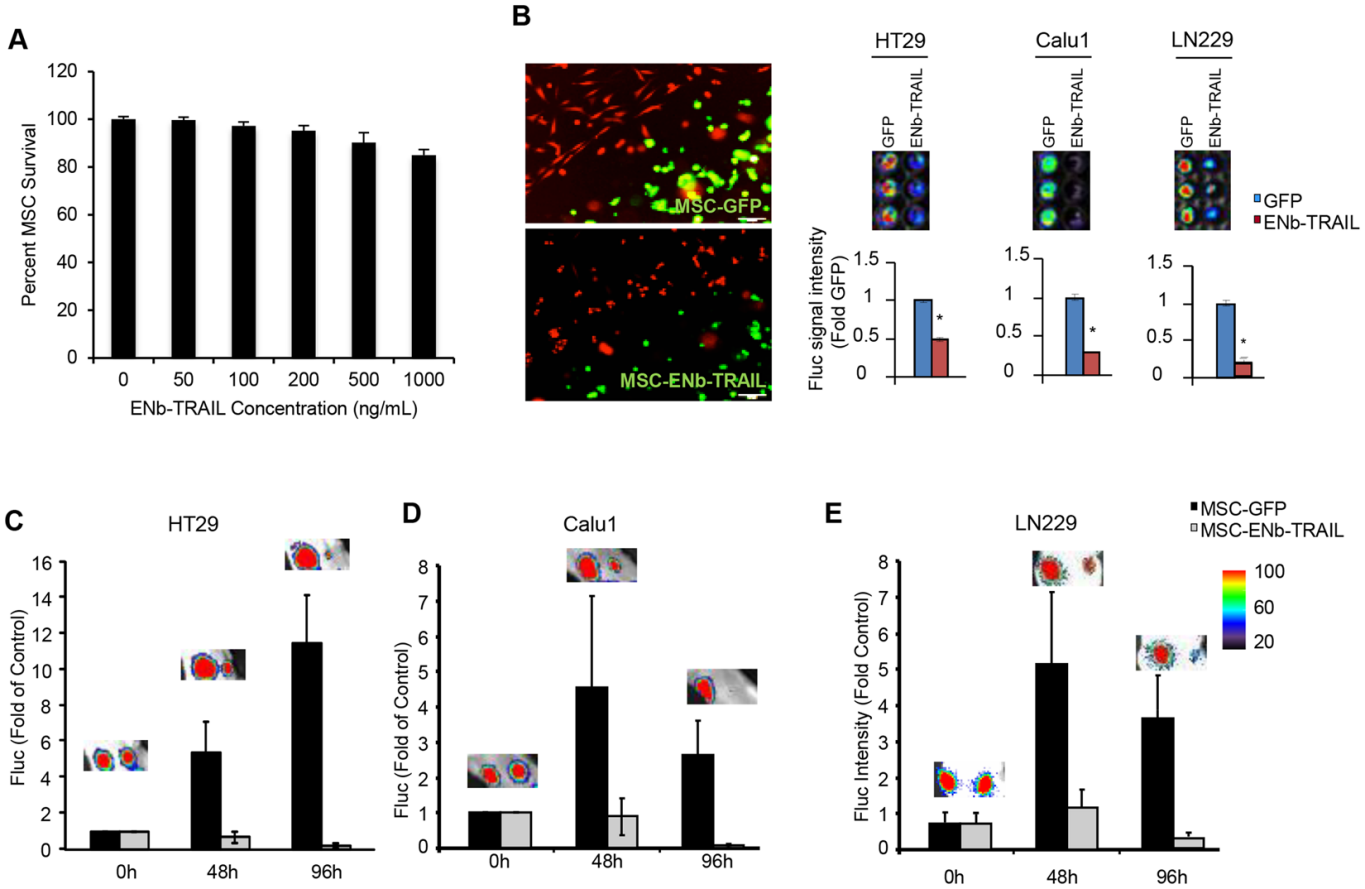
Therapeutic stem cell delivered ENb-TRAIL is effective *in vitro* and *in vivo*. (**A**) Plot showing the response of human MSC to different concentrations of ENb-TRAIL. (**B**) Tumor cells were engineered with FmC and tumor cell viability was visualized by Fluc bioluminescence imaging 48 h post co-culture with MSC-GFP or MSC-ENb-TRAIL-IRES-GFP. Left panel: high magnification fluorescence images of co-culture; Right panel: Fluc bioluminescence images of co-culture and the quantification of Fluc signals. *P < 0.001 determined by unpaired *t* test. Error bars indicate SD. (**C**–**E**) Mice were subcutaneously implanted with HT29-FmC cells (**C**), Calu1-FmC cells (**D**) or LN229-FmC (**E**) cells and ad-mixed with MSC (GFP or ENb-TRAIL-IRES-GFP) and the fate of tumor cells was followed by Fluc bioluminescence imaging. Representative Fluc bioluminescence images and quantitation is shown as fold of 0 h post implantation. Data are mean +/−SD.

Short drug half-life and dose-limiting nonspecific toxicities are major challenges for systemic delivery of anti-cancer agents^26^. As human mesenchymal stem cells (MSCs) are attractive candidates for targeted cellular delivery of therapeutics^27^, we sought to test the efficacy of MSC delivered ENb-TRAIL *in vivo*. We screened the response of human MSC to varying doses of ENb-TRAIL and show that MSC are resistant to ENb-TRAIL (Figs 4A and S7). Similarly, ENb-TRAIL did not significantly reduce viability of human astrocytes in culture (Fig. S8). We therefore engineered MSC to constitutively secrete ENb-TRAIL. *In vitro* co-culture of MSC-ENb-TRAIL- IRES-GFP or MSC-GFP with tumor cells engineered to express the dual imaging marker Fluc-mCherry (FmC) showed that MSC delivered ENb-TRAIL has therapeutic efficacy (Fig. 4B). *In vivo* we implanted an ad-mixture of labeled colon cancer cells HT29-FmC (Fig. 4C), lung cancer cells Calu1-FmC (Fig. 4D) and glioblastoma cells, LN229-FmC (Fig. 4E) with MSC-ENb-TRAIL or MSC-GFP and followed tumor cell fate by Fluc bioluminescent imaging. A significant decrease of tumor volumes was observed in all three tumor cell lines implanted with MSC-ENb-TRAIL as compared to the controls as early as 48 h post implantation. By 96 h, a decrease of over 90% of Fluc signal was observed in these cell lines as compared to the time of implantation (Fig. 4C–E). These data show that MSC delivered ENb-TRAIL is effective *in vivo* in ENb and TRAIL non-responsive tumor cells.

**Figure 5:**
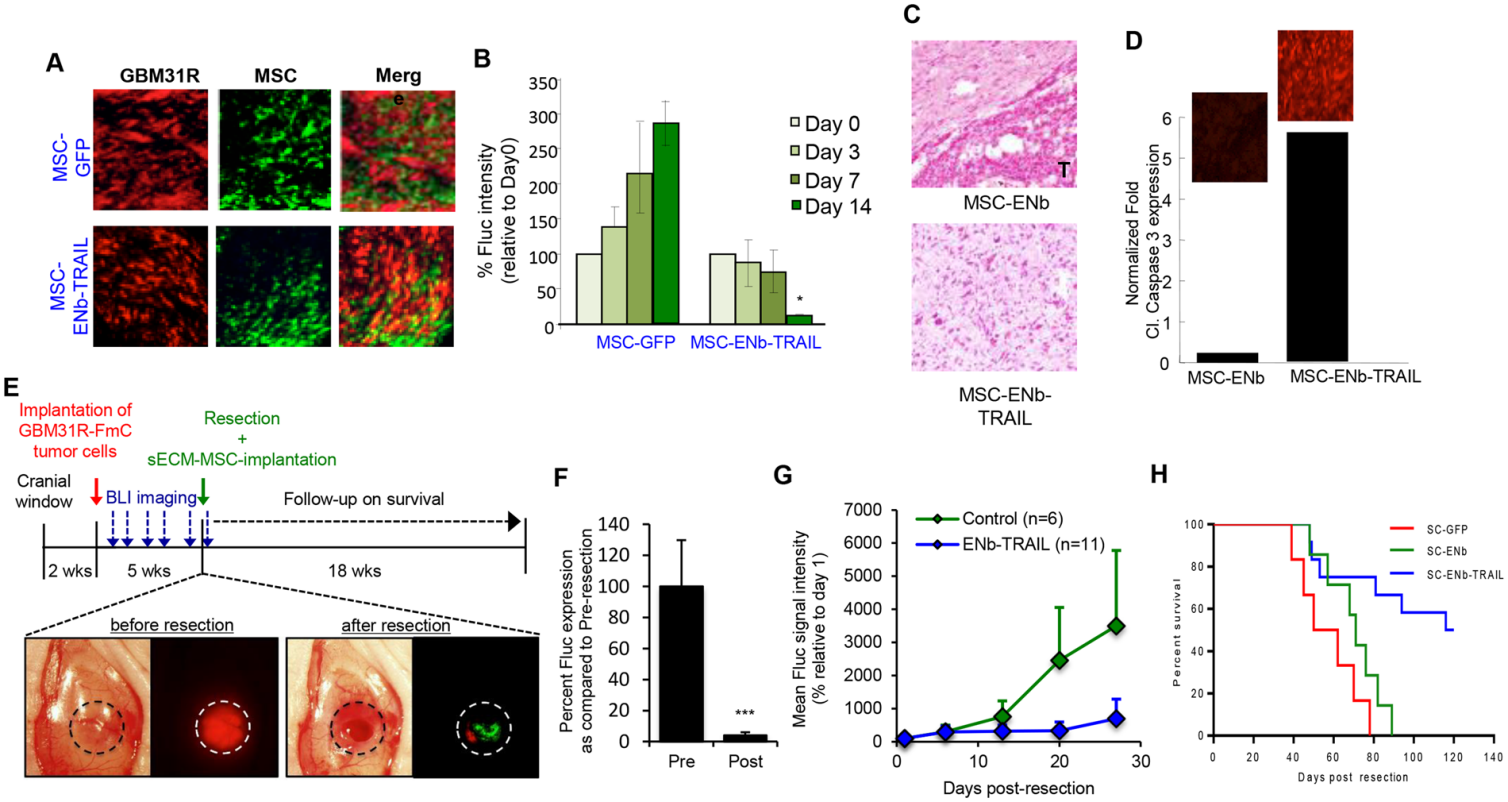
MSC delivered ENb-TRAIL has anti-tumor effects in mouse tumor model of GBM resection. (**A**) Photomicrographs from mice brains on Day 3 post-implantation of GBM31R-FmC (red) and engineered MSC (green). (**B**) Plot showing changes in tumor volumes of GBM31R-FmC cells as compared to Day 0 following treatment with MSC-GFP or MSC-ENb-TRAIL-IRES-GFP (**C**) H&E stain from mouse brain sections obtained 21 days post MSC administration. (**D**) Plot showing changes in cleaved Caspase 3 expression on brain sections harvested 3 days post administration of MSC-GFP or MSC-ENb-TRAIL (inset) representative photomicrographs revealing expression of Cl. Caspase 3 (**E**) Top: Timeline of GBM *in vivo* experiment and MSC-ENb-TRAIL efficacy in the GBM31R resection mouse model. Bottom left: Light and fluorescence photomicrographs of an intracranial implanted GBM31R-FmC tumor in a cranial window prior to resection; Bottom right: Light and merged fluorescence photomicrograph of the tumor resection cavity (red; remaining GBM31R-FmC tumor cells) before and after implantation of sECM encapsulated MSC-GFP cells (green). (**F**) Analysis of pre- and post-resection Fluc intensity (normalized to pre-resection). (**G**) Plot demonstrating the GBM31R-FmC tumor growth post-resection in different treatment groups over time (Control vs. ENb-TRAIL at day 27, p = 0.066). (**H**) Kaplan-Meier survival curve analysis of different treatment groups (SC-ENb-TRAIL vs. SC-GFP p < 0.0001; SC-ENb-TRAIL vs. SC-ENb p < 0.0019). *P < 0.05, **P < 0.005 and ***P < 0.0001, determined by unpaired *t* test.

In order to validate the above findings in mouse models that mimic the clinical scenario of tumor growth and treatment with surgical resection, we chose a patient derived recurrent primary glioblastoma line, GBM31R (isolated after the first-line resection and treatment with temozolomide) that has low-level surface expression of EGFR and DR4 and high level of DR5 and responds to ENb-TRAIL treatment in a dose dependent manner (Fig. S9A–C). GBM31R cells were engineered to express the dual imaging marker FmC (GBM31R-FmC) (Fig. S9D) and an admixture of GBM31R-FmC and MSC-GFP or MSC-ENb-TRAIL (3:1 ratio) were implanted into the right cerebral hemisphere. Fluorescence imaging on brain sections from mice on day 3 post administration showed the presence of both cell types (Fig. 5A). A significant reduction in tumor cell growth as indicated by FLuc signal intensity was seen in mice that received MSC-ENb-TRAIL as compared to control treatment (Fig. 5B). H&E stains of brain sections additionally confirmed the reduction of tumor burden in mice treated with MSC-ENb-TRAIL as compared to control mice (Fig. 5C). Immuno-staining of brain sections revealed an increased expression of cleaved caspase-3 in tumors brains treated with MSC-ENb-TRAIL as compared to controls (Fig. 5D). To exclude ENb-TRAIL induced toxicity in normal brain, MSC-ENb-TRAIL were implanted into the right cerebral hemisphere of naïve mice followed by assessment for ENb-TRAIL induced cell killing in the normal brain surrounding the implantation site. Immuno-staining of brain sections did not show any expression of cleaved caspase-3 in the GFAP expressing normal brain cells (Fig. S10).

To mimic the clinical scenario of surgical GBM debulking, we developed an orthotopic mouse model of GBM31R resection and evaluated the efficacy of sECM encapsulated MSC delivered ENb-TRAIL in this model. Mice bearing GBM31R-FmC tumors (Figs 5E and S9E) underwent fluorescence guided tumor resection (Fig. 5E) which resulted in significant tumor reduction (Fig. 5F). To avoid washout of MSC in the tumor resection cavity, engineered MSC were encapsulated in synthetic extracellular matrices (sECM) and subsequently implanted in the tumor resection cavity (Fig. 5E). Bioluminescent imaging showed that mice treated with sECM-MSC-ENb-TRAIL had significantly less tumor re-growth as compared to the sECM-MSC-GFP treated group (Fig. 5G) resulting in a significant increase in the median survival of sECM-MSC-ENb-TRAIL treated mice (118 days vs 73.5 days) (Fig. 5H). Together, these data reveal that MSC delivered ENb-TRAIL specifically kills tumor cells in the brain and has therapeutic efficacy in a mouse tumor model of glioblastoma that mimics the clinical scenario of tumor growth and resection.

## Discussion

In this study, we characterized the molecular mechanism underlying the anti-tumor activity of ENb-TRAIL and evaluated its therapeutic efficacy in various cell lines from different cancer types which are inherently non-responsive to EGFR and DR4/5 targeted monotherapies. We demonstrate that ENb-TRAIL engages both EGFR and DR5 simultaneously to form *EGFR*-ENb-TRAIL-*DR5* complex, resulting in DR5 clustering at the plasma membrane and subsequent induction of caspase-mediated apoptosis *in vitro* and in mouse tumor models.

**Figure 6:**
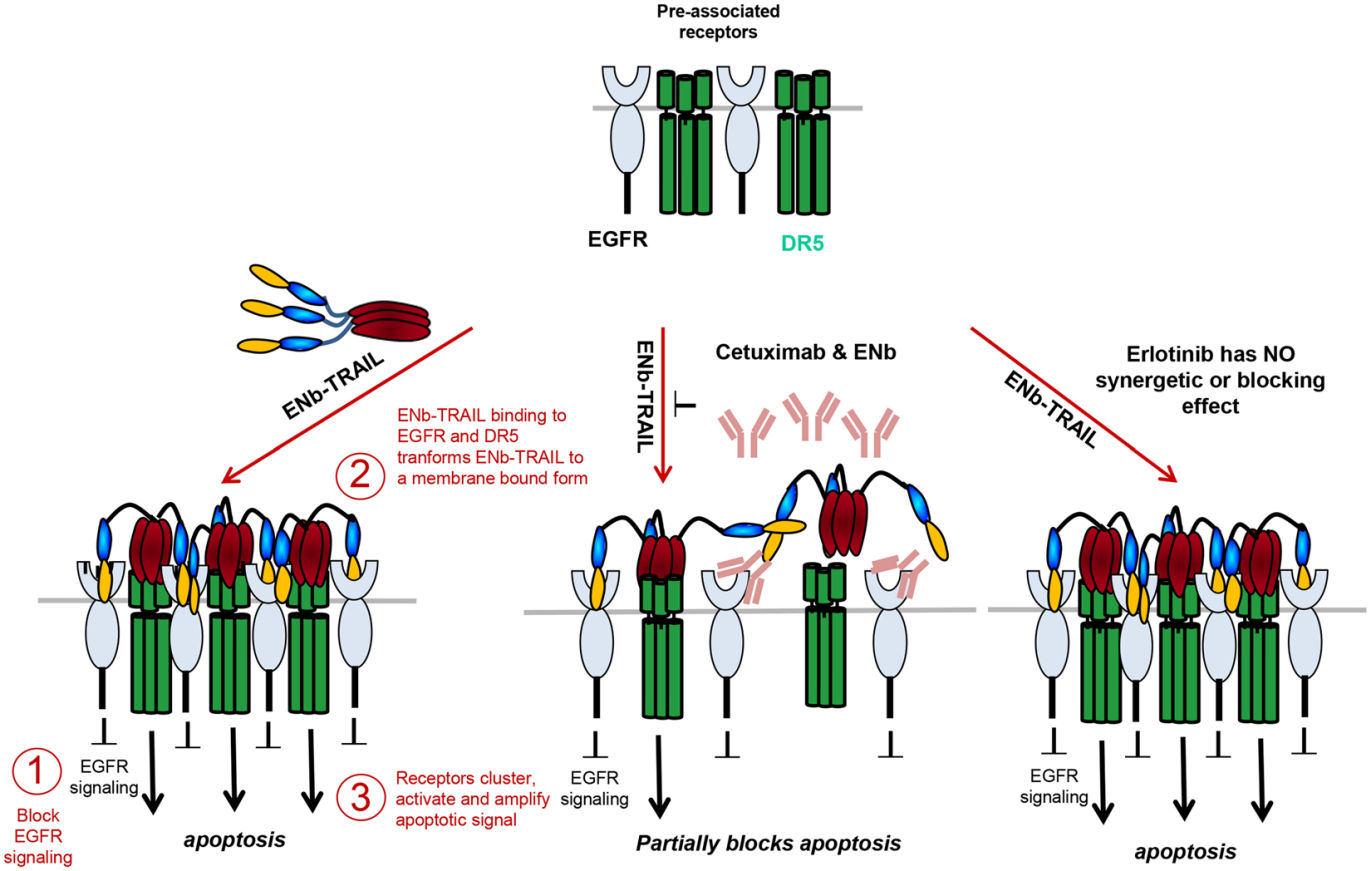
Model of ENb-TRAIL function. EGFR and DR5 are co-engaged at the surface of tumor cells. Trimerized ENb-TRAIL binds simultaneously to EGFR and DR5 leading to receptor clustering and amplification of apoptotic signal. Blocking EGFR with Cetuximab or ENb abolishes ENb-TRAIL binding to EGFR and subsequent activation of apoptosis. EGFR tyrosine kinase inhibitor, Erlotinib inhibits EGFR and AKT activity but does not influence activation of apoptosis by ENb-TRAIL.

EGFR is frequently overexpressed and/or mutated in cancer, resulting in increased activation of cell proliferation and pro-survival pathways thus rendering EGFR an excellent target for cancer therapy^28^. While targeting over-expressed and mutated EGFR on tumor cells is a promising approach it has provided very limited clinical benefit in the setting of glioblastoma^29^. There are several EGFR mediated signaling resistance mechanisms that have become evident that potentially reduce efficacy of DR targeted therapeutic agents. For example, EGFR signaling has been shown to operate via activation of PI3K-AKT pathway and we and others have shown that phosphorylated active AKT is a potent inhibitor of apoptosis through its ability to phosphorylate pro-apoptotic proteins^30–34^. Furthermore, signaling cross talk between the EGFR pathway and the DR pathway has been previously shown by studies in which inhibition of EGFR or PI3K/AKT sensitizes cells to TRAIL-induced apoptosis^32^. Therefore, it is critical to develop strategies to simultaneously target cell proliferation and death pathways in tumor cells. Our results reveal that ENb-TRAIL is directly involved in activating DRs in addition to blocking EGFR thereby priming tumor cells for DR mediated apoptosis (Fig. 6). Furthermore, our studies also reveal that the combined treatment with ENb and TRAIL was not sufficient in inducing apoptosis in TRAIL and ENb unresponsive tumor cells, suggesting that ENb-TRAIL binding to EGFR and DR5 was not only suppressing EGFR pathway and initiating apoptotic pathways, but had additional functions enabling ENb-TRAIL to strongly induce apoptosis in different tumor cell types. These data indicate that the bi-functionality of ENb-TRAIL is not fully recapitulated by a combination treatment of tumor cells with ENb and TRAIL. Our findings are in line with previous observations that although the efficacy of different mono-targeting therapies is often improved by their combination^35, 36^, the versatility of bi- or multi-targeting formats provide opportunities for the enhancement of cancer therapies via simultaneous engagement of more than one cell surface receptor. Furthermore, our findings revealed that EGFR blocking studies with Cetuximab or ENb reduced ENb-TRAIL induction of apoptosis and the levels of *EGFR*-ENb-TRAIL-*DR5* complex suggesting that ENb-TRAIL binding to EGFR is necessary for the EGFR-ENb-TRAIL-DR5 complex formation and optimum activation of DR5 (Fig. 6).

Caspases are categorized as initiator or effector caspases, based on their position in apoptotic signaling cascades. Caspases-8 is an initiator caspase, acts apically in cell death pathways and are activated when adaptor proteins interact with the prodomains and promote caspase dimerization^37, 38^. In contrast, caspase-3 and 7 are effector caspases and following cleavage mediated by an initiator caspase, they act directly on specific cellular substrates to dismantle the cell. In our studies, the activity of caspase 3 evaluates the final outcome of ENb-TRAIL induced apoptosis, and the activations of caspase-8 measured by cleaved caspase 8 are consistent with the caspase 3 activities suggesting that no other intrinsic pathways are involved in ENb-TRAIL induced apoptosis.

Previous studies have shown that a significant number of tumor cells express both DR4 and DR5 and each of these receptors can initiate apoptosis independently. However DR5 is expressed highly on tumor cells than DR4^39^ and has therefore been shown to contribute more than DR4 to TRAIL-induced apoptosis in cancer cells that express both DR4 and DR5^40^. Consistent with these findings, our studies show that three different tumor types shared similar DR5 cell surface expression levels and have only minimal or no cell surface expression of DR4. Furthermore, our DR4/5 knock down and DR4 overexpression data suggests that DR5 is the major player in mediating ENb-TRAIL-induced apoptosis. The differential response of tumor lines HT29, Calu1, and LN229 to ENb-TRAIL is not sufficiently reflected by the levels of cell surface EGFR and DR5 alone. These differences in sensitivity to ENb-TRAIL between tumor lines may possibly be the result of individual, cell-line dependent heterogeneity.

Our FRET observations provide direct evidence that EGFR and DR5 are co-localized and in close proximity to allow ENb-TRAIL to bind to both EGFR and DR5 simultaneously. Previous studies have shown that both TRAIL and DR4/5 do not require internalization for formation of the death-inducing signaling complex (DISC), activation of caspase-8, or transmission of an apoptotic signal, and blocking DR5 endocytosis does not inhibit apoptosis^41, 42^. Our studies did not address whether ENb-TRAIL binding induces EGFR and DR5 endocytosis and further studies are warranted to understand if ENb-TRAIL leads to receptor endocytosis and whether this correlates with increased apoptosis.

The *in vitro* response of tumor cell lines to ENb-TRAIL is not directly predictive of the *in vivo* response, therefore it is necessary to test the response of tumor cells to ENb-TRAIL *in vivo*. We and others have previously shown that NSC and MSC migrate extensively toward tumors and therefore have an enormous therapeutic potential as gene delivery vehicles^43–47^. Most of the cancer cell lines used in this study were specifically chosen based on their unresponsiveness to death receptor agonist, TRAIL. As such, the use of MSC-TRAIL on tumors generated from these tumor lines would not have any significant therapeutic effect *in vivo*. In this study, we armed human MSC with ENb-TRAIL and showed that transgene expression is maintained *in vitro* and *in vivo* over a period of time without affecting stem cell properties. In the subcutaneous admixture experiments of GBM, colon and lung cancer models, the therapeutic efficacy of MSC-ENb-TRAIL was prominent as seen by the reduction in BLI intensities that are representative of tumor volumes. Interestingly, a more significant effect of ENb-TRAIL on tumor cell growth was observed *in vivo* than in culture conditions, which could be attributed to sustained delivery of ENb-TRAIL via MSC and has been shown in previous studies^23, 48^. Additionally, this could also be due to the amplification of EGFR and subsequent *in vivo* gain of EGFR-dependence of tumor cells in mouse tumor models^49^. These findings demonstrate the therapeutic antitumor potential of on-site delivered bispecific therapeutic molecules via engineered stem cells. However, further preclinical characterization of the pharmacokinetic properties of stem cell released molecules *in vivo* will be required before these findings are translatable into a clinical setting in different tumor types.

In an effort to translate stem cell based ENb-TRAIL into clinical settings in brain tumor patients, we tested the efficacy of ENb-TRAIL in primary patient derived GBM cells *in vitro* and *in vivo*. Given the central role tumor resection plays in GBM therapy^50^, we have previously developed mouse GBM models of resection and shown that encapsulation of stem cells is necessary to prevent rapid “wash- out” of stem cells in the tumor resection cavity^51^. Our data reveal that sECM encapsulated MSC-ENb-TRAIL has therapeutic efficacy in a mouse tumor model of glioblastoma that mimics the clinical scenario of tumor growth and resection. The testing of ENb-TRAIL efficacy in clinically relevant tumor models using proper delivery vehicles is critical to ultimately develop effective therapies for a broad spectrum of tumors.

In conclusion, our mechanistic characterization and preclinical evaluation of ENb-TRAIL in various types of tumor cells provides a new basis for future studies of multifunctional targeted molecules in cancer therapy. The observed therapeutic efficacy of ENb-TRAIL on tumors that are unresponsive to EGFR and DR4/5 mono-targeted therapies in this study encourages additional research with regards to the simultaneous targeting of multiple receptor-mediated signaling pathways and holds promise to aid in overcoming mono-targeted therapeutic resistance.

## Materials and Methods

### Antibodies and Reagents

The following primary antibodies and reagents were used in this study: Antibodies against phospho-AKT(Ser-473), AKT, Caspase 8, cleaved Caspase 3, EGFR, cleaved PARP, phospho-S6 ribosomal protein(Ser-235/236), S6 ribosomal protein, phospho-p-44/42MAPK(ERK1/2) (Thr202/Tyr204), and p-44/42MAPK(ERK1/2) (Cell Signaling); anti-phospho-EGFR(Tyr-1068) (Abcam); anti-α Tubulin (Sigma); anti-DR5 (Santa Cruz); Cetuximab (ImClone Systems); Erlotinib (Selleck Chemicals); human recombinant EGF (R&D Systems); anti-human CD261 (DR4)PE and anti-human CD262 (DR5)PE(eBioscience).

### Cell lines

Human colorectal cancer cell lines HT29 and HCT116 and established human glioma cell line LN229 were grown in DMEM supplemented with 10% (vol/vol) FBS and 1% (vol/vol) penicillin/streptomycin. Human lung cancer cell lines HCC827 and Calu1 were grown in RPMI1640 supplemented with 10% (vol/vol) FBS and 1% (vol/vol) penicillin/streptomycin. Patient derived primary GBM cell lines GBM6 and GBM31R were grown in neural basal medium (Invitrogen) supplemented with 3 mM L-Glutamine, B27 supplement, N2 supplement, 2µg/ml heparin, 20 ng/ml EGF, and 20 ng/ml bFGF as described previously^52^. Human bone marrow-derived MSCs were grown as described previously^27^.

### shRNA knock down of DR4 or DR5

Cells were transduced with lentiviruses, PLKO-1 shDR4, shDR5 or control shRNAs (Open Biosystems) and cell viability and caspase activity on tumor cells expressing shDR4, shDR5 and controls post-ENb-TRAIL treatment were performed as described above. All experiments were performed in triplicate.

### *In vivo* assessment of MSC-ENb-TRAIL efficacy in colon, lung, and GBM cancer cells

SCID mice (3 week of age, Charles River Laboratories, Wilmington, MA) were implanted subcutaneously with HT29-mCherryFluc, Calu1-mCherryFluc, LN229-mCherryFluc cells (1 × 10^5^; n = 4/group) in a 1:2 mixture of MSC-GFP or MSC-ENb-TRAIL-IRES-GFP cells and tumor cell fate was imaged by Fluc activity as described^23^.

### *In vivo* assessment of MSC-ENb-TRAIL efficacy in a clinically relevant mouse model of GBM tumor resection

To examine the *in vivo* efficacy of MSC-delivered ENb-TRAIL in a primary TRAIL-resistant, EGFR expressing GCS mouse intracranial xenograft resection model, female SCID mice (8–9 weeks of age, 20–25 g in weight) were immobilized on a stereotactic frame 14 days prior to tumor implantation. Using a SZX10 stereo-microscope system (Olympus), a small circular portion of the skull covering the right cerebral hemisphere (~7 mm in diameter) was removed in order to create a cranial window for subsequent GCS implantation and fluorescence-guided surgical tumor debulking. Two weeks later the mice were again immobilized on a stereotactic frame, the previously established cranial window was exposed and GBM31R-mCherryFluc cells (2 × 10^5^ cells per mouse) were superficially implanted into the right cerebral cortex (2.5-mm lateral from bregma, 0.5-mm deep) of 25 mice. In the following weeks, tumor growth was non-invasively monitored via bioluminescence imaging (BLI) of the tumor cells Fluc signal activity as previously described^45^. Approximately 5 weeks after tumor implantation, Fluc signal intensity had increased significantly and mice then underwent fluorescence-guided tumor resection followed by implantation of stem cells into the resection cavity as previously described^51^. Groups were as follows: MSC-GFP (n = 7), MSC-ENb-IRES-GFP (n = 8) and MSC-ENb-TRAIL-IRES-GFP (n = 17). To prevent washout of stem cells from the resection cavity by cerebrospinal fluid (CSF), MSCs were encapsulated in biodegradable ECM by mixing 2 × 10^5^ of respective MSCs per mouse with Hystem (6 μl) and matrix cross-linker Extralink (4 μl) approximately 20 minutes prior to implantation in order to allow gel-formation. Mice were imaged for Fluc signal intensity post resection in order to assess tumor debulking. Successful resection was defined as at least a 10-fold postoperative reduction of BLI signal. Of the above groups n = 23 mice met these criteria (MSC-GFP: n = 6; MSC-ENb-IRES-GFP: n = 6; MSC-ENb-TRAIL- IRES-GFP: n = 11) and only these mice were then further followed for survival and sacrificed when neurological symptoms became apparent. Kaplan-Meier curves were generated for survival analysis.

All methods were performed in accordance with the relevant guidelines and regulations and all *in vivo* procedures were approved by the Sub-committee on Research Animal Care at MGH.

## Electronic supplementary material

Supplementary Figure legends

## References

[1] Nicholson RI, Gee JM, Harper ME (2001). EGFR and cancer prognosis. Eur J Cancer.

[2] Selvaggi G (2004). Epidermal growth factor receptor overexpression correlates with a poor prognosis in completely resected non-small-cell lung cancer. Annals of oncology: official journal of the European Society for Medical Oncology/ESMO.

[3] Shinojima N (2003). Prognostic value of epidermal growth factor receptor in patients with glioblastoma multiforme. Cancer research.

[4] Thatcher N (2005). Gefitinib plus best supportive care in previously treated patients with refractory advanced non-small-cell lung cancer: results from a randomised, placebo-controlled, multicentre study (Iressa Survival Evaluation in Lung Cancer). Lancet.

[5] Martinelli E, De Palma R, Orditura M, De Vita F, Ciardiello F (2009). Anti-epidermal growth factor receptor monoclonal antibodies in cancer therapy. Clinical and experimental immunology.

[6] Lievre A (2006). KRAS mutation status is predictive of response to cetuximab therapy in colorectal cancer. Cancer research.

[7] Yonesaka K (2011). Activation of ERBB2 signaling causes resistance to the EGFR-directed therapeutic antibody cetuximab. Science translational medicine.

[8] Kobayashi S (2005). EGFR mutation and resistance of non-small-cell lung cancer to gefitinib. The New England journal of medicine.

[9] Engelman JA (2007). PF00299804, an irreversible pan-ERBB inhibitor, is effective in lung cancer models with EGFR and ERBB2 mutations that are resistant to gefitinib. Cancer Res.

[10] Wiezorek J, Holland P, Graves J (2010). Death receptor agonists as a targeted therapy for cancer. Clinical cancer research: an official journal of the American Association for Cancer Research.

[11] Bellail AC, Olson JJ, Yang X, Chen ZJ, Hao C (2012). A20 ubiquitin ligase-mediated polyubiquitination of RIP1 inhibits caspase-8 cleavage and TRAIL-induced apoptosis in glioblastoma. Cancer discovery.

[12] Voortman J, Resende TP, Abou El Hassan MA, Giaccone G, Kruyt FA (2007). TRAIL therapy in non-small cell lung cancer cells: sensitization to death receptor-mediated apoptosis by proteasome inhibitor bortezomib. Molecular cancer therapeutics.

[13] Kaler P, Galea V, Augenlicht L, Klampfer L (2010). Tumor associated macrophages protect colon cancer cells from TRAIL-induced apoptosis through IL-1beta-dependent stabilization of Snail in tumor cells. PloS one.

[14] Schaefer G (2011). A two-in-one antibody against HER3 and EGFR has superior inhibitory activity compared with monospecific antibodies. Cancer cell.

[15] Castoldi R (2013). A novel bispecific EGFR/Met antibody blocks tumor-promoting phenotypic effects induced by resistance to EGFR inhibition and has potent antitumor activity. Oncogene.

[16] Wang X (2007). *In vitro* efficacy of immuno-chemotherapy with anti-EGFR human Fab-Taxol conjugate on A431 epidermoid carcinoma cells. Cancer biology & therapy.

[17] Wu G (2007). Molecular targeting and treatment of an epidermal growth factor receptor-positive glioma using boronated cetuximab. Clinical cancer research: an official journal of the American Association for Cancer Research.

[18] Williams SC (2013). Small nanobody drugs win big backing from pharma. Nature medicine.

[19] Roovers RC (2011). A biparatopic anti-EGFR nanobody efficiently inhibits solid tumour growth. International journal of cancer. Journal international du cancer.

[20] Vosjan MJ (2012). Nanobodies targeting the hepatocyte growth factor: potential new drugs for molecular cancer therapy. Molecular cancer therapeutics.

[21] Maussang D (2013). Llama-derived single variable domains (nanobodies) directed against chemokine receptor CXCR7 reduce head and neck cancer cell growth *in vivo*. The Journal of biological chemistry.

[22] Oliveira S, Heukers R, Sornkom J, Kok RJ, van Bergen En Henegouwen PM (2013). Targeting tumors with nanobodies for cancer imaging and therapy. Journal of controlled release: official journal of the Controlled Release Society.

[23] van de Water JA (2012). Therapeutic stem cells expressing variants of EGFR-specific nanobodies have antitumor effects. Proceedings of the National Academy of Sciences of the United States of America.

[24] Li S (2005). Structural basis for inhibition of the epidermal growth factor receptor by cetuximab. Cancer cell.

[25] Schmitz KR, Bagchi A, Roovers RC, van Bergen en Henegouwen PM, Ferguson KM (2013). Structural evaluation of EGFR inhibition mechanisms for nanobodies/VHH domains. Structure.

[26] Kelley SK (2001). Preclinical studies to predict the disposition of Apo2L/tumor necrosis factor-related apoptosis-inducing ligand in humans: characterization of *in vivo* efficacy, pharmacokinetics, and safety. The Journal of pharmacology and experimental therapeutics.

[27] Sasportas LS (2009). Assessment of therapeutic efficacy and fate of engineered human mesenchymal stem cells for cancer therapy. Proceedings of the National Academy of Sciences of the United States of America.

[28] Citri A, Yarden Y (2006). EGF-ERBB signalling: towards the systems level. Nat Rev Mol Cell Biol.

[29] Roth P, Weller M (2014). Challenges to targeting epidermal growth factor receptor in glioblastoma: escape mechanisms and combinatorial treatment strategies. Neuro-oncology.

[30] Panner A (2010). Ubiquitin-specific protease 8 links the PTEN-Akt-AIP4 pathway to the control of FLIPS stability and TRAIL sensitivity in glioblastoma multiforme. Cancer Res.

[31] Puduvalli VK (2005). TRAIL-induced apoptosis in gliomas is enhanced by Akt-inhibition and is independent of JNK activation. Apoptosis.

[32] Shrader M (2007). Gefitinib reverses TRAIL resistance in human bladder cancer cell lines via inhibition of AKT-mediated X-linked inhibitor of apoptosis protein expression. Cancer research.

[33] Zhai B (2014). Inhibition of akt reverses the acquired resistance to sorafenib by switching protective autophagy to autophagic cell death in hepatocellular carcinoma. Mol Cancer Ther.

[34] Zhu Y, Shah K (2014). Multiple lesions in receptor tyrosine kinase pathway determine glioblastoma response to pan-ERBB inhibitor PF-00299804 and PI3K/mTOR dual inhibitor PF-05212384. Cancer biology & therapy.

[35] Engelman JA (2008). Effective use of PI3K and MEK inhibitors to treat mutant Kras G12D and PIK3CA H1047R murine lung cancers. Nature medicine.

[36] Xu L (2012). Combined EGFR/MET or EGFR/HSP90 inhibition is effective in the treatment of lung cancers codriven by mutant EGFR containing T790M and MET. Cancer Res.

[37] Boatright KM (2003). A unified model for apical caspase activation. Molecular cell.

[38] Wachmann K (2010). Activation and specificity of human caspase-10. Biochemistry.

[39] Ashkenazi A (2002). Targeting death and decoy receptors of the tumour-necrosis factor superfamily. Nature reviews. Cancer.

[40] Kelley RF (2005). Receptor-selective mutants of apoptosis-inducing ligand 2/tumor necrosis factor-related apoptosis-inducing ligand reveal a greater contribution of death receptor (DR) 5 than DR4 to apoptosis signaling. The Journal of biological chemistry.

[41] Kohlhaas SL, Craxton A, Sun XM, Pinkoski MJ, Cohen GM (2007). Receptor-mediated endocytosis is not required for tumor necrosis factor-related apoptosis-inducing ligand (TRAIL)-induced apoptosis. The Journal of biological chemistry.

[42] Ichikawa K (2001). Tumoricidal activity of a novel anti-human DR5 monoclonal antibody without hepatocyte cytotoxicity. Nature medicine.

[43] Aboody KS (2000). Neural stem cells display extensive tropism for pathology in adult brain: evidence from intracranial gliomas. Proceedings of the National Academy of Sciences of the United States of America.

[44] Sasportas LS (2009). Assessment of therapeutic efficacy and fate of engineered human mesenchymal stem cells for cancer therapy. Proceedings of the National Academy of Sciences.

[45] Shah K (2008). Bimodal viral vectors and *in vivo* imaging reveal the fate of human neural stem cells in experimental glioma model. The Journal of neuroscience: the official journal of the Society for Neuroscience.

[46] Tang Y (2003). *In vivo* tracking of neural progenitor cell migration to glioblastomas. Human gene therapy.

[47] Yamazoe T (2015). Potent tumor tropism of induced pluripotent stem cells and induced pluripotent stem cell-derived neural stem cells in the mouse intracerebral glioma model. International journal of oncology.

[48] Hingtgen SD, Kasmieh R, van de Water J, Weissleder R, Shah K (2010). A novel molecule integrating therapeutic and diagnostic activities reveals multiple aspects of stem cell-based therapy. Stem Cells.

[49] Pandita A, Aldape KD, Zadeh G, Guha A, James CD (2004). Contrasting *in vivo* and *in vitro* fates of glioblastoma cell subpopulations with amplified EGFR. Genes Chromosomes Cancer.

[50] Chang SM (2005). Patterns of care for adults with newly diagnosed malignant glioma. Jama.

[51] Kauer, T. M., Figueiredo, J. L., Hingtgen, S. & Shah, K. Encapsulated therapeutic stem cells implanted in the tumor resection cavity induce cell death in gliomas. *Nat Neurosci* (2012). 10.1038/nn.3019 PMC3601490 22197831

[52] Wakimoto H (2009). Human glioblastoma-derived cancer stem cells: establishment of invasive glioma models and treatment with oncolytic herpes simplex virus vectors. Cancer research.

