# Supplementary material for: Bi-specific molecule against EGFR and death receptors simultaneously targets proliferation and death pathways in tumors
Source: Sci Rep. 2017 Jun 1;7:2602. doi: 10.1038/s41598-017-02483-9 (PMC5454031; doi:10.1038/s41598-017-02483-9)
Supplement: Supplementary file 1 — Supplementary Figure legends [file 41598_2017_2483_MOESM1_ESM.pdf]

Supplementary Figures

Bi-specific molecule against EGFR and death receptors simultaneously targets proliferation and death pathways in tumor cells

Yanni Zhu[1,2,3], Nicole Bassoff [1,2,3], Clemens Reinshagen[1,2,3], Deepak Bhere[1,2,3], Michal O. Nowicki[5], , Sean E. Lawler[5], Jérémie Roux[6], and Khalid Shah[1,2,3,4,7*]

Fig. S1. (A) Diagrammatic representation of the ENb-TRAIL construct. SS: signal sequence. (B) Table showing the known genetic information of RTK pathway in the different cancer cell lines. (C) Western blot analysis of EGFR, DR4, and DR5 expression in the cell lines shown in B

Fig. S2. Western blot analysis of EGFR signaling in Erlotinib treated HT29 cells

Fig. S3. (A) Quantification of cleaved Caspase-8 expression data shown in Fig.1E. (B) Live cell imaging of LN229 reveals the dynamics of ENb-TRAIL induced apoptosis compared to combined treatment with ENb plus TRAIL. Left panel: Density distribution of cell death times for LN229 cells dying after ENb-TRAIL treatment (red) compared to ENb + TRAIL co-treatment (green). Right panel: Fraction of surviving LN229 cells at 24hrs after ENb-TRAIL treatment (red) compared to ENb + TRAIL co-treatment (green). 250 cells were analyzed per well per condition. (C) Cell viability of HT29 and LN229 cells in response to 24h treatment with TRAIL(T) vs Erlotinib (E) plus TRAIL(E+T) (*upper panel*) or ENb-TRAIL (NT) vs Erlotinib plus ENb-TRAIL(E+NT) (*lower panel*).

Fig. S4. (A) Caspase 3/7 assay analysis of ENb effect on ENb-TRAIL induction of apoptosis. Cells were pretreated with ENb (100nM) for 30 min and then treated with ENb-TRAIL (100ng/ml) for 8h and apoptosis was measured by caspase 3/7 assay. * $P<0.05$ and ** $P< 0.005$ determined by unpaired *t* test. Error bars indicate SD. (B) Quantification of cleaved Caspase-8 expression shown in Fig. 2B.

Fig. S5. (A) Quantification of DR5 protein immunoprecipitated by EGFR shown in Fig. 2D. (B) Confocal image of transient co-expression of DR5-CFP and EGFR-YFP in 293T cells without ENb-TRAIL (upper

Supplementary Figure 1

A

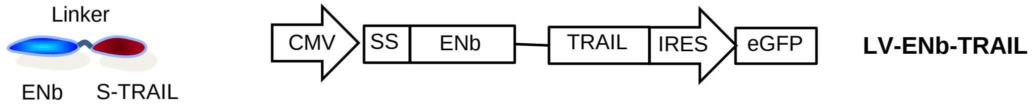

B

	HT29	HCT116	HCC827	Calu1	LN229	GBM6
EGFR	WT	WT	ΔE746-A750	WT	WT	WT
RAS		KRASmt		KRASmt		
RAF	BRAF (V600E)					
RTKs					ERBB2/3	

C

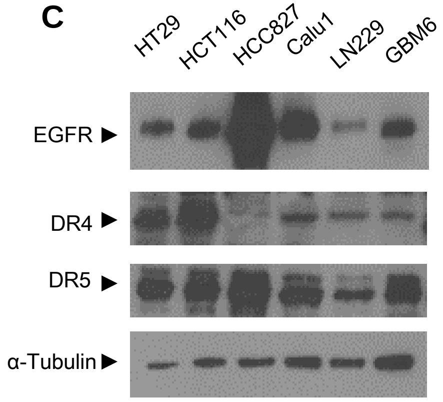

Supplementary Figure 2

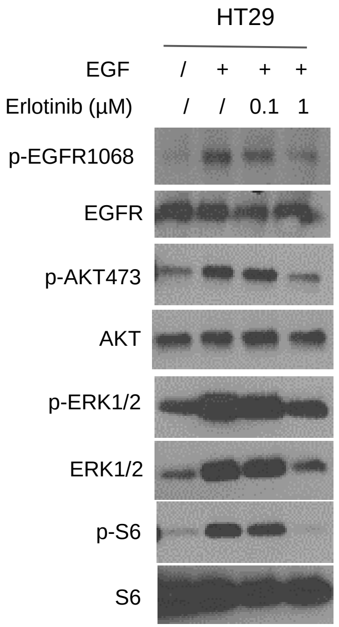

Supplementary Figure 3

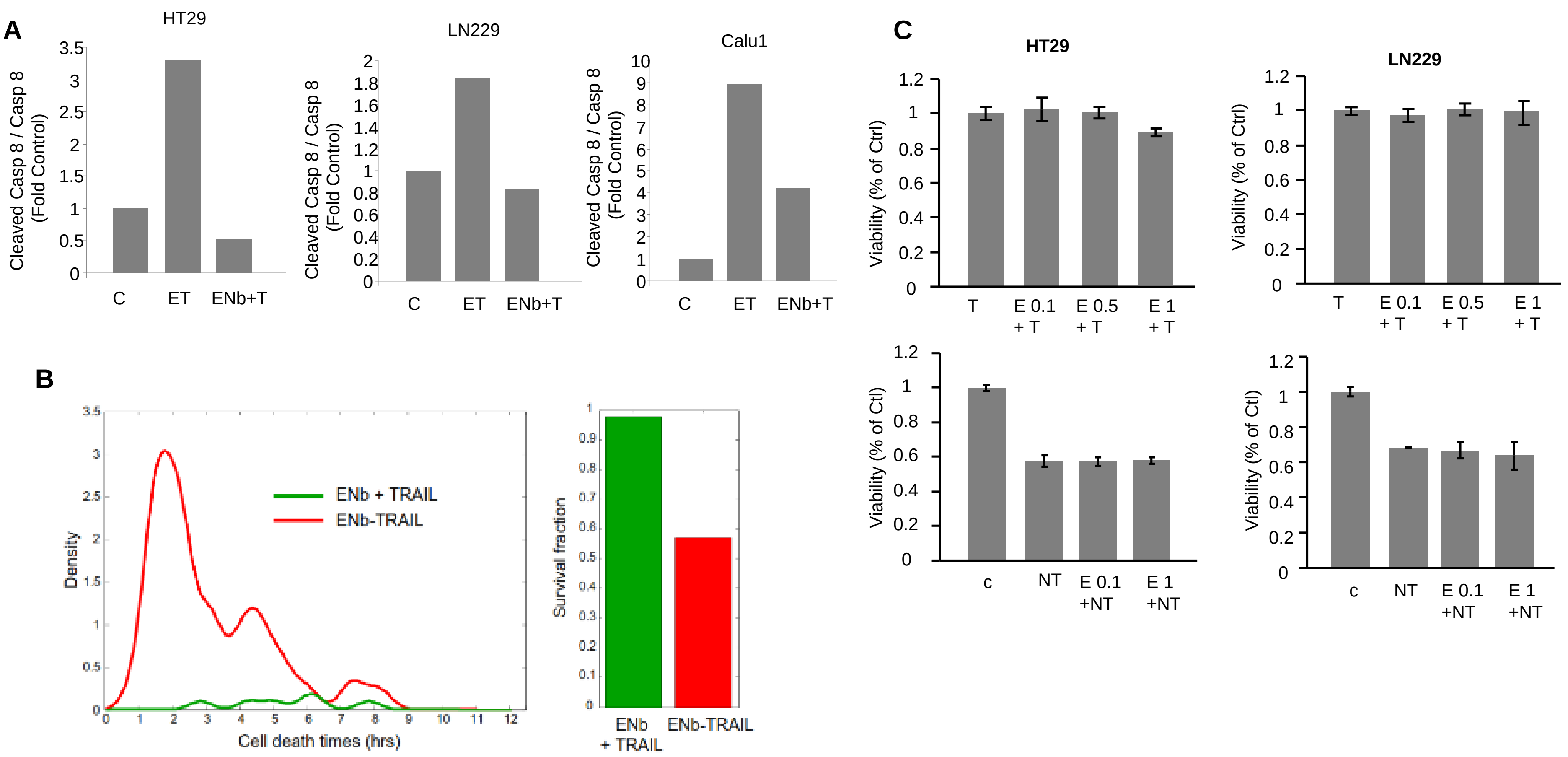

Supplementary Figure 4

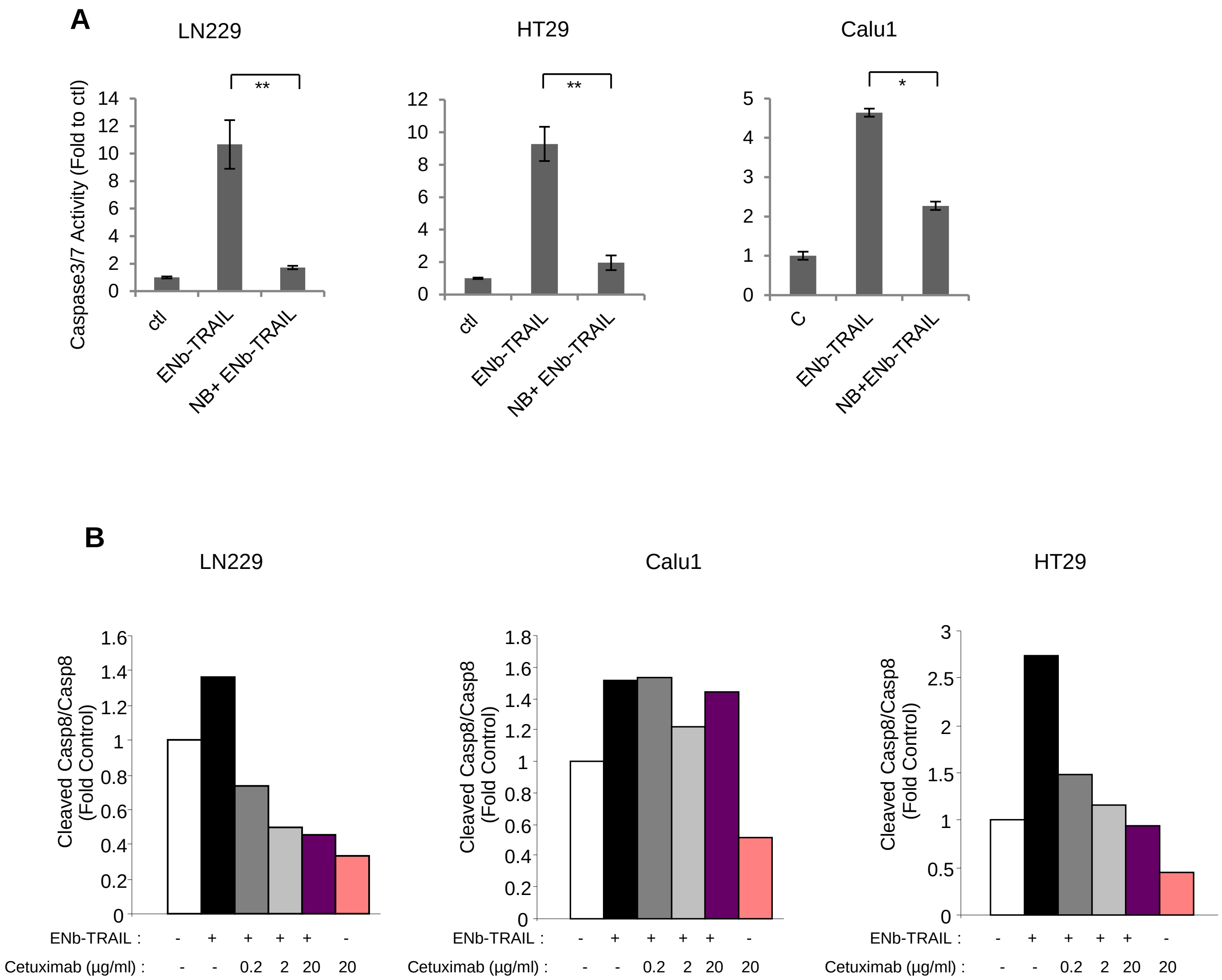

Supplementary Figure 5

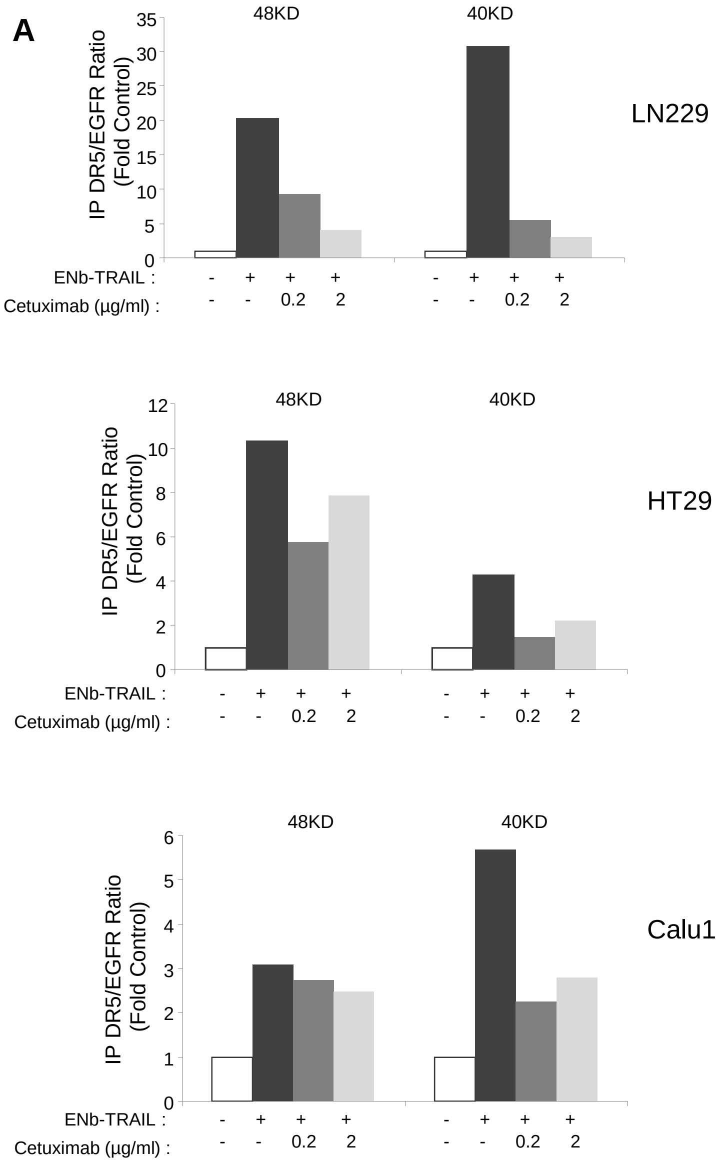

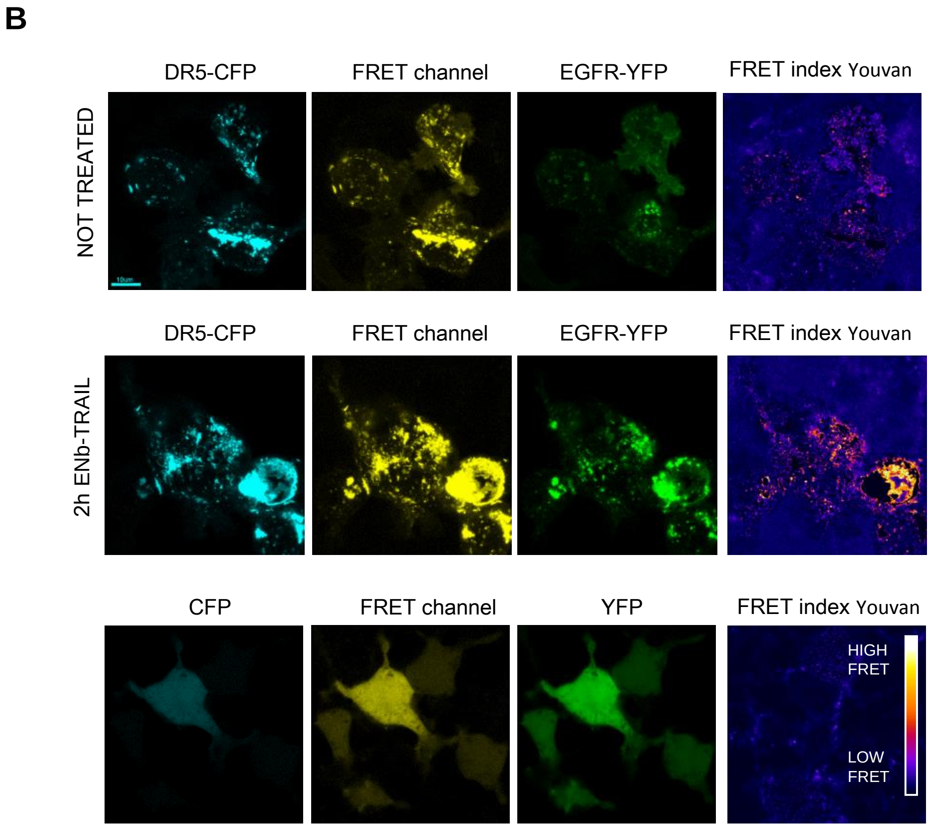

Supplementary Figure 6

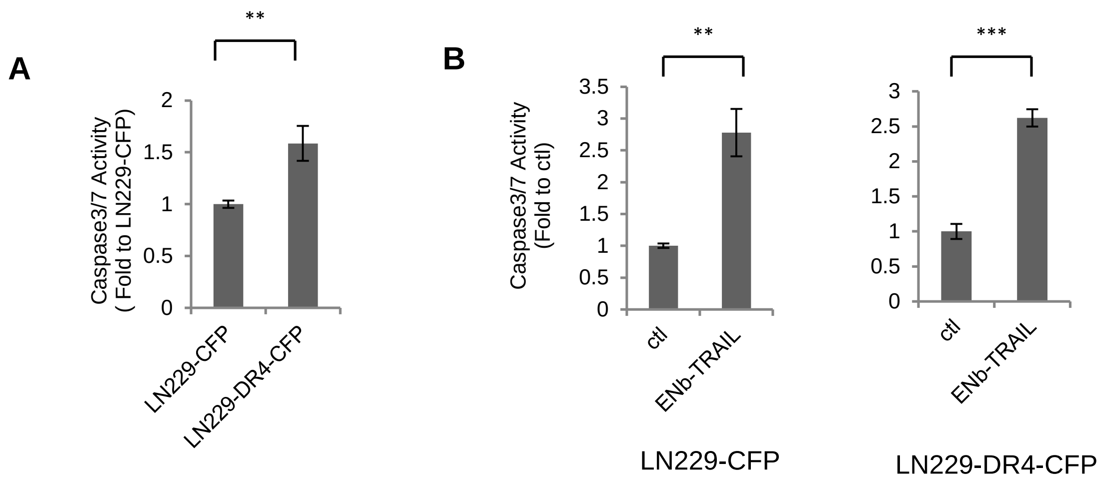

Supplementary Figure 7

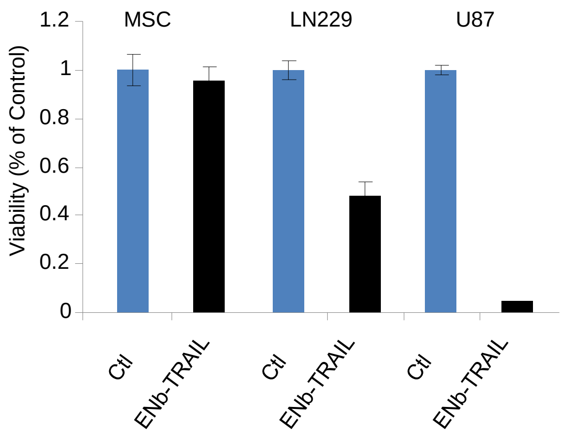

Supplementary Figure 8

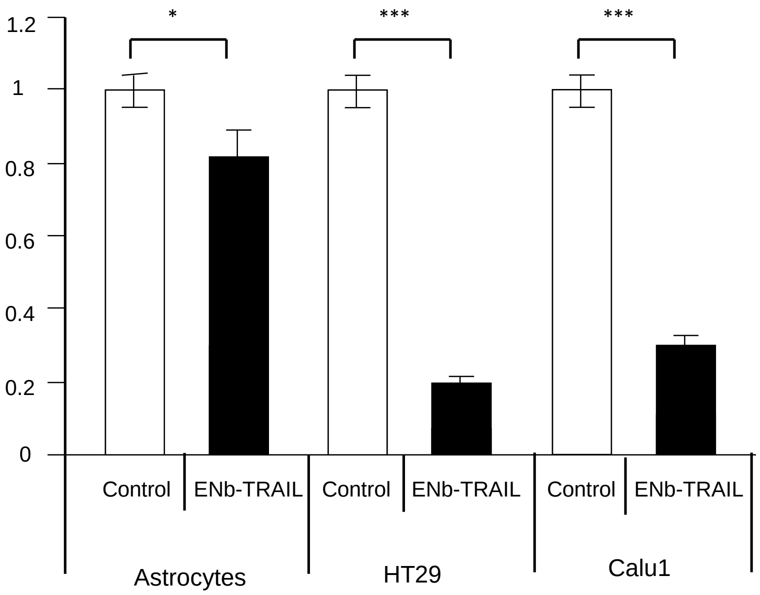

Supplementary Figure 9

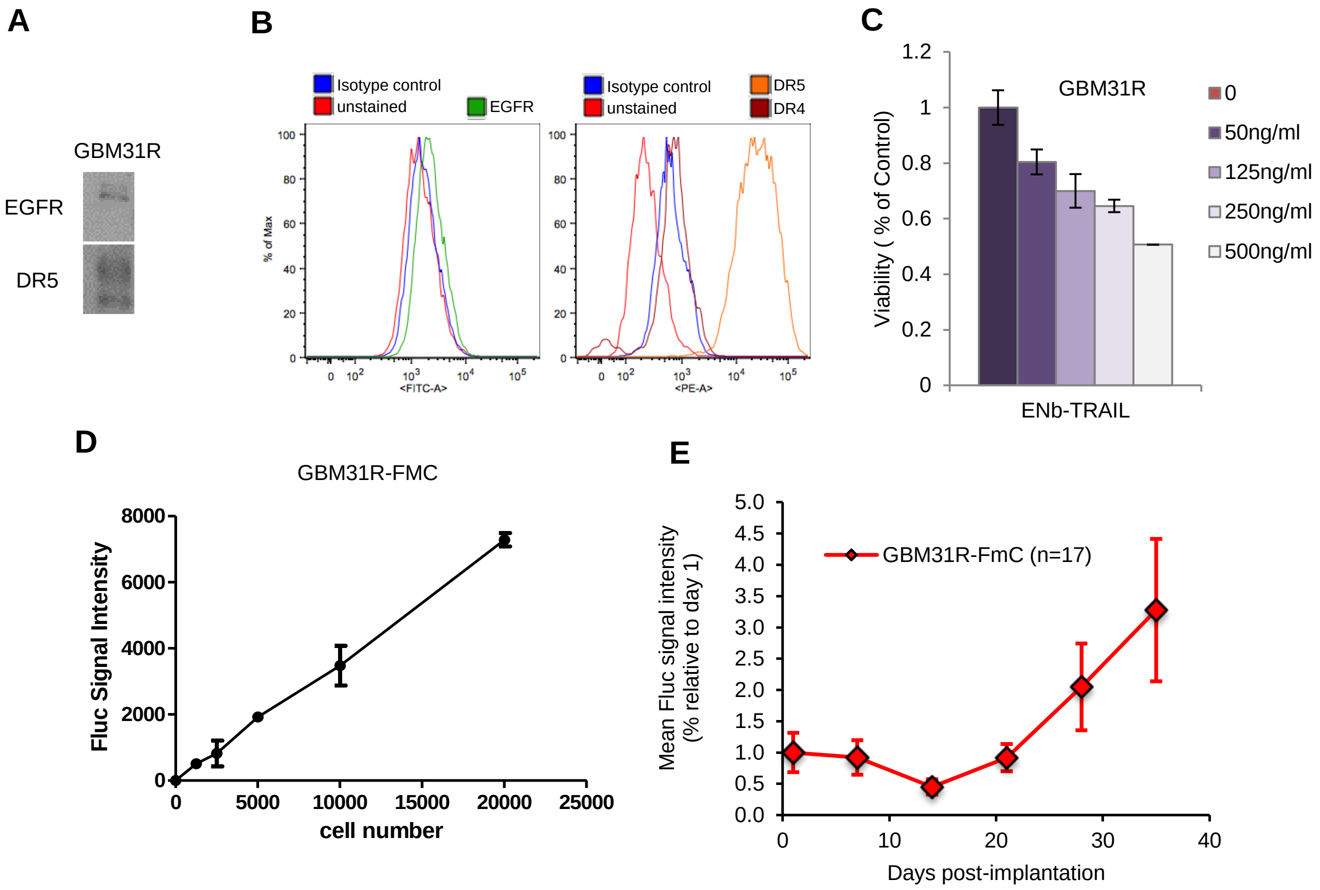

Supplementary Figure 10

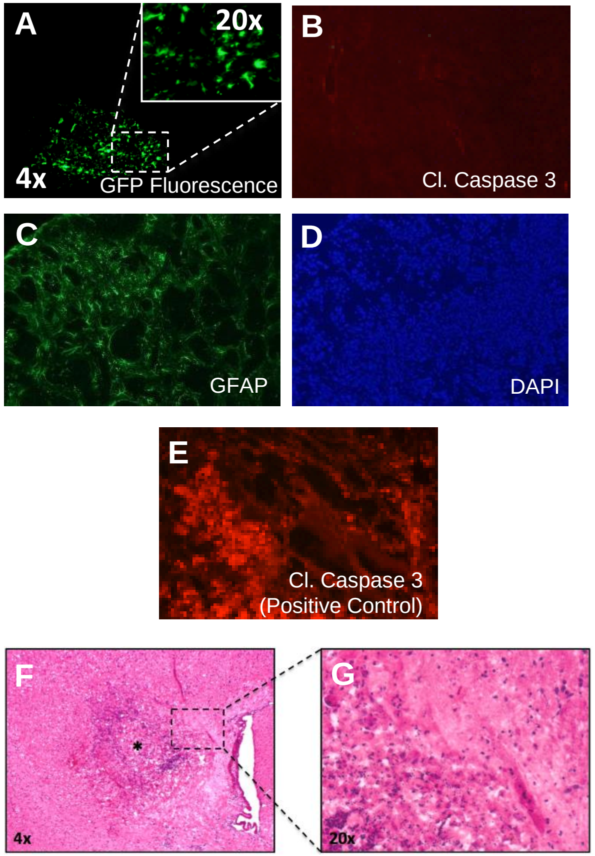

Supplementary Figure 11

Fig. 1C – uncropped western

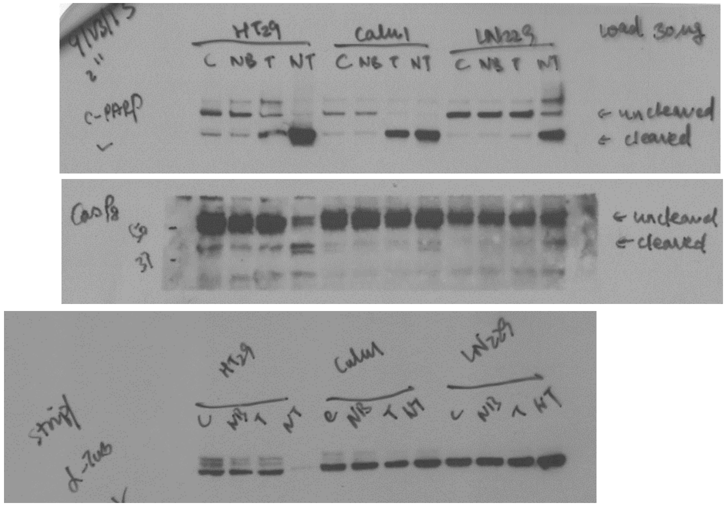

Fig. 1D – uncropped western

ENb-treated

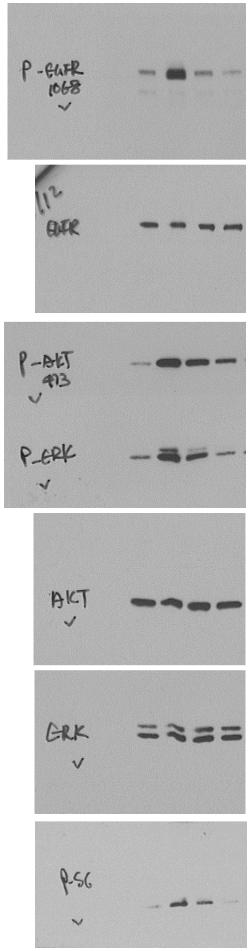

Fig. 1E – uncropped western

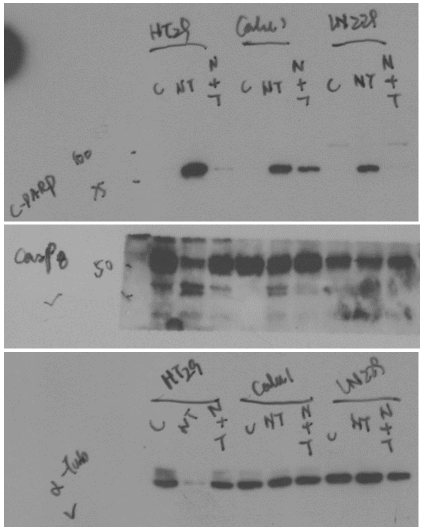

Supplementary Figure 12

Fig. 2B – uncropped western

LN229

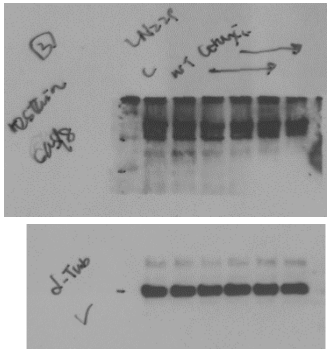

HT29

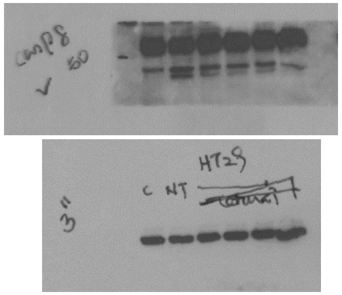

Calu1

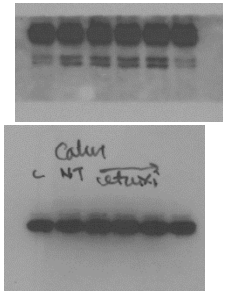

Fig. 2D – uncropped western

LN229

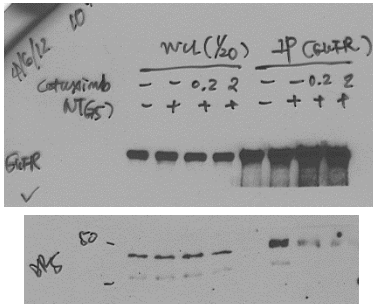

HT29

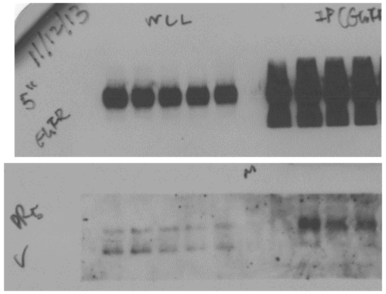

Calu1

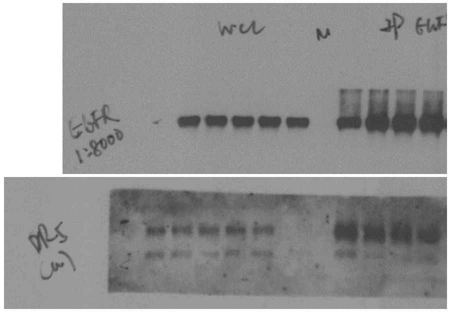

Fig. 3A – uncropped western

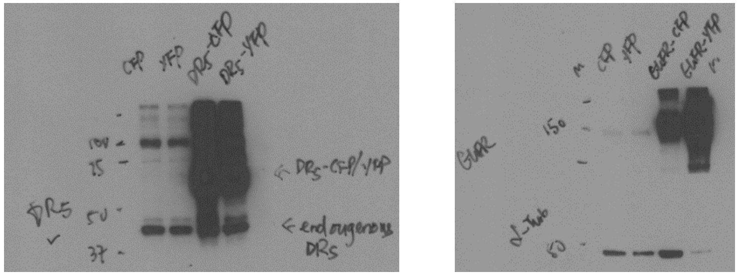

panel) or 2h after ENb-TRAIL treatment (middle panel), and co-expression of CFP and YFP in 293T cells (bottom panel).

Fig. S6. Caspase3/7 assay analysis of LN229-DR4-CFP cells treated with ENb-TRAIL for 24h. (A) Caspase3/7 activity analysis of LN229 cells transduced with CFP versus DR4-CFP. (B) Caspase 3/7 activity analysis of ENb-TRAIL treatment in LN229-CFP and LN229-DR4-CFP cells. * P<0.05, ** P< 0.005 and *** P=0.0001 determined by unpaired *t* test. Error bars indicate SD.

Fig. S7. Cell viability of human MSC and glioma cells in response to 48h treatment with ENb-TRAIL.

Fig. S8. Cell viability of human astrocytes NHA and tumor cells in response to 48h treatment with ENb-TRAIL. * P<0.05, ** P< 0.005 and *** P=0.0001 determined by unpaired *t* test. Error bars indicate SD.

Fig. S9. (A) Western blot analysis of EGFR and DR5 expression in patient derived glioblastoma line, GBM31R; (B) FACS analysis showing low-level expression of EGFR and DR4 and high level of DR5 surface expression of GBM31R. (C) Cell viability of GBM31R in response to different doses of ENb-TRAIL treatment for 24h. (D) Plot showing the correlation between Fluc signal intensity and GBM31R-FmC cell number. (E) Plot demonstrates GBM31R tumor growth post-implantation.

Fig. S10. Photomicrographs from mice brain sections harvested 5 days post administration of MSC-ENb-TRAIL-IRES-GFP showing the presence of MSC (A). Brain sections were analyzed for the expression of cleaved Caspase-3 and GFAP expression and stained for DAPI. Photomicrographs showing negative cleaved Caspase-3 staining (B) GFAP expression (C) and DAPI staining (D) in normal brain surrounding the MSC-ENb-TRAIL implantation site. (E) Positive control showing cleaved Caspase-3 staining on tumor cells in the brain. (F-G) Low and high magnification images of H&E staining showing the site of MSC-ENb-TRAIL implantation (indicated by *) and the normal brain surrounding the implanted cells.

Fig. S11. Figure showing uncropped western blots from Fig. 1.

Fig. S12. Figure showing uncropped western blots from Fig. 2&3.

Supporting Methods

Bi-specific molecule against EGFR and death receptors simultaneously targets proliferation and death pathways in tumor cells

Yanni Zhu[1,2,3], Nicole Bassoff [1,2,3], Clemens Reinshagen[1,2,3], Deepak Bhere[1,2,3], Michal O. Nowicki[5], , Sean E. Lawler[5], Jérémie Roux[6], and Khalid Shah[1,2,3,4,7*]

Lentiviral transductions and engineering stable cell lines: Lentivral packaging was performed by transfection of 293T cells as previously described (1). MSCs were transduced with lentiviral vectors (LV-GFP or LV-ENb-TRAIL-IRES-GFP) at multiplicity of infection (M.O.I) = 4 in growth medium containing 8µg/ml protamine sulfate (Sigma-Aldrich). Cancer cells were transduced with LV-Pico2-Fluc-mCherry at M.O.I of 2 in medium-containing protamine sulfate and selected by puromycin (1µg/ml) in culture. GFP or mCherry expression was visualized by fluorescence microscopy.

Cell viability and caspase assays: Tumor cells were plated in 96-well plates and treated with different doses of Cetuximab, Erlotinib, ENb, TRAIL or ENb-TRAIL for 24h. Cell viability was measured using an ATP-dependent luminescent reagent (CellTiterGlo, Promega) and caspase activity was determined using a DEVD-aminoluciferin assay (CaspaseGlo 3/7, Promega) according to manufacturer's instructions. All experiments were performed in triplicate.

Live cell imaging: LN229 cells were grown on glass-bottom 96-well plates (Matrical Bioscience) for 24h in complete medium. ENb-TRAIL or ENb and TRAIL were added and time is depicted at t=0. Time-lapse microscopy movies were recorded with an Operetta (Perkin Elmer) fluorescence microscope equipped with an environmental chamber at 10x magnification with frames every 5 minutes for 24 hours. Cell death time was determined in ImageJ by visual inspection, which enabled an identification of the first frame of apoptotic morphology change.

Engineering DR4-CFP cell lines: LN229 cells were transduced with lentiviral vectors (LV-CFP or LV-DR4-

CFP) at multiplicity of infection (M.O.I) = 2 in growth medium containing 8μg/ml protamine sulfate (Sigma-Aldrich). CFP or DR4-CFP expressions were visualized by fluorescence microscopy and CFP positive cells were sorted by flow cytometry.

Co-immunoprecipitation and immunoblotting: Following treatment, cells were washed with cold PBS twice, then lysed with cold RIPA buffer (20 mM Tris-HCl pH8.0, 137mM NaCl, 10% glycerol, 1% NP-40, 0.1% SDS, 0.5% Na-deoxycholate, 2mM EDTA pH8.0) with protease and phosphatase inhibitors (Phosphatase Inhibitor Cocktail I and Phosphatase Inhibitor Cocktail II from Sigma-Aldrich). Cells were scraped into 1.5ml microtubes and centrifuged at 4℃, 16,000g for 10 minutes. Supernatant protein concentrations were determined using a Bio-Rad protein assay kit. Supernatants were immunoprecipitated with the indicated antibody for 4 hours at 4℃, and washed with cold PBS three times. 6X SDS-sample buffer was added to the washed samples, boiled for 3 minutes and resolved by SDS-PAGE gel. For blotting whole cell lysate, 10-30 μg of protein was resolved on SDS-PAGE gel, transferred to nitrocellulose membrane and probed with primary antibodies.

Flow cytometry analysis of cell surface receptors: Cells were dissociated, washed and re-suspended in 0.5% BSA, 2mM EDTA solution in PBS. Cells were stained with Alexa Fluor 488-conjugated anti-human EGFR (BioLegend), PE-conjugated anti-human DR4 or PE-conjugated anti-human DR5 monoclonal antibodies (eBioscience) in solution at 4℃ for 30 min. Rinses were performed with 0.5% BSA, 2mM EDTA at 4℃. Alexa Fluor 488 or PE-conjugated isotype specific IgGs were used as control. Flow cytometry was performed using FACSAria II (BD) cell sorter and data was analyzed using FlowJo software.

Co-culture of tumor cells and engineered MSCs: Fluc-mCherry engineered tumor cells ($2x10^3$ per well) were co-cultured with either MSC-ENb-TRAIL-IRES-GFP or MSC-GFP cells ($1x10^4$ cells) in 96 well plate. 48 hours later, the relative number of tumor cells was determined by Fluc bioluminescence imaging as described.

Fluorescent protein fusion constructs and FRET imaging: The LV-DR4/5-CFP and LV-EGFR-YFP constructs were generated by ligation of CFP and YFP cDNAs into NheI/NheI digested LV-DR4/5-GFP-

RLUC or LV-EGFR-GFP-RLUC respectively (2). 293T cells (ATCC) were grown at 37°C in a humidified incubator in DMEM with 5% CO_2. Cells were seeded at 20,000 cells/well in 8-well Nunc™ Lab-Tek™ II slides and transfected with fusion protein constructs using Lipofectamine 2000 (Invitrogen) according to manufacturer's recommendations. Time-lapse image acquisition was performed 24 hours post transfection. Cells were treated with ENb-TRAIL (100ng/ml) immediately prior to image acquisition with a Zeiss LSM710 confocal microscope equipped with an environmental chamber and a high resolution 40x lens. The donor channel was set up as ext.458nm/em.460-510nm, acceptor channel ext.514nm/em550-650nm, and the FRET channel ext.458nm/em550-650nm using lasers at the lowest possible power. Analysis and quantification of FRET signal was performed using Zeiss ZEN 3.0 software and Microsoft Excel.

References:

1. Shah K, *et al.* (2008) Bimodal viral vectors and in vivo imaging reveal the fate of human neural stem cells in experimental glioma model. *J Neurosci* 28(17):4406-4413.

2. Arwert E, *et al.* (2007) Visualizing the dynamics of EGFR activity and antiglioma therapies in vivo. *Cancer Res* 67(15):7335-7342.
